# Functional Interaction between Right Parietal and Bilateral Frontal Cortices during Visual Search Tasks Revealed Using Functional Magnetic Imaging and Transcranial Direct Current Stimulation

**DOI:** 10.1371/journal.pone.0093767

**Published:** 2014-04-04

**Authors:** Amanda Ellison, Keira L. Ball, Peter Moseley, James Dowsett, Daniel T. Smith, Susanne Weis, Alison R. Lane

**Affiliations:** 1 Department of Psychology, Durham University, Durham, United Kingdom; 2 Department of Psychology, Carl von Ossietzky Universität Oldenburg, Oldenburg, Germany; University Medical Center Goettingen, Germany

## Abstract

The existence of a network of brain regions which are activated when one undertakes a difficult visual search task is well established. Two primary nodes on this network are right posterior parietal cortex (rPPC) and right frontal eye fields. Both have been shown to be involved in the orientation of attention, but the contingency that the activity of one of these areas has on the other is less clear. We sought to investigate this question by using transcranial direct current stimulation (tDCS) to selectively decrease activity in rPPC and then asking participants to perform a visual search task whilst undergoing functional magnetic resonance imaging. Comparison with a condition in which sham tDCS was applied revealed that cathodal tDCS over rPPC causes a selective bilateral decrease in frontal activity when performing a visual search task. This result demonstrates for the first time that premotor regions within the frontal lobe and rPPC are not only necessary to carry out a visual search task, but that they work together to bring about normal function.

## Introduction

The neural networks involved in visual attention have been broadly defined as involving frontoparietal regions for directing attention and the eye to locations within the visual field [1]. Using a variety of behavioural tasks these regions have been further defined as the frontal eye fields (FEF), supplementary eye fields (SEF) and posterior parietal cortex (PPC) [2]–[7] of the right hemisphere.

The visual search paradigm has shown itself to be a useful tool when investigating the role of these specific regions in issues relating to stimulus features, the identification of targets and distractors, spatial localisation, and the deployment and allocation of attention. Whilst functional magnetic resonance imaging (fMRI) studies have shown that right FEF and right PPC (rPPC) are co-activated in conjunction visual search tasks [3], [4], [8], neurostimulation studies using transcranial magnetic stimulation (TMS) have sought to define the critical involvement of these two areas in such tasks. It would seem that both are critically involved and not merely co-activated [9]–[13], with some evidence that the two regions have separable roles. For example, rPPC is only involved when the target appears in non-primed, or unpredictable space (as is left FEF), whilst right FEF is involved regardless of target position [13].

One currently unresolved issue is whether or not there is a functional connectivity between FEF and PPC within the right hemisphere. There is evidence that FEF is involved in visual search tasks earlier than PPC [14], leading to the idea that a contingency may exist between these areas. Having previously used event related TMS to demonstrate co-operation between the brain regions of rPPC and lateral occipital cortex [15], and rPPC and V5 [16], we were unable to demonstrate a similar effect between rPPC and right FEF [17]. This may be due to compensation by left FEF and/or the conservative nature of the technique. However, using transcranial direct current stimulation (tDCS) we demonstrated dissociations in the communication network between these regions [18]. Specifically, Ball et al. (2013) reported that right FEF has a more transient role than rPPC in conjunction visual search tasks, such that cathodal stimulation (which decreases cortical excitability) over rPPC impaired search performance, whereas anodal stimulation (which increases excitability) had no effect. The large electrodes used in this, and the present, study do not preclude the possibility that right tempoparietal junction (TPJ) activity has also been modulated, an area which has also been found to be involved in the reorientation of visual attention using TMS [19].

Whilst this work sheds light on the nuances of PPC and FEF behaviour in steady state and dynamic processing, it cannot add evidence to the question as to whether or not these regions work concurrently or independently to bring about competent visual search performance. We propose to build upon our previous knowledge to investigate this question using cathodal tDCS to decrease activity in rPPC, and fMRI to examine the consequential distal excitability changes in other regions of the brain during a conjunction visual search task.

## Materials and Methods

### Ethics Statement

This study was specifically approved by the Durham University Ethics Advisory Committee. Participants gave their signed informed consent in accordance with the Declaration of Helsinki and could withdraw at any time.

### Participants

20 participants (12 male) with normal or corrected to normal vision from Durham University took part in this experiment (age range 21 to 56 years, mean age 27.95, *SD* = 7.72, all right handed). Participant selection complied with the current guidelines for tDCS and fMRI research. One participant had to be excluded from the analysis of the fMRI data due to extensive head movement in the scanner.

### Transcranial Direct Current Stimulation

Two rubber electrodes were placed in two sponge pouches (7 cm×5 cm) which had been soaking in a physiologically active saline solution. A rubber strap was used to hold the two electrodes in place on the scalp. tDCS was applied using a Magstim Eldith DC stimulator for 15 minutes at a current intensity of 1.5 mA, an intensity which has been reported to induce changes in neuronal activity lasting up to one hour [20]–[23]. Stimulation protocol complied with the current safety guidelines for tDCS [24]. There were two stimulation conditions (Cathodal and Sham) and one stimulation site (rPPC). Thus, there were two experimental sessions separated by one week, a session in which cathodal stimulation was applied and one in which sham was applied. The order of the sessions was counter-balanced across the participants.

In the cathodal stimulation condition the cathode was placed over rPPC and the anode electrode was placed above the participant’s left eye in accordance with our previous work (Ball et al., 2013). In the sham condition the electrode placement was the same but participants received stimulation for only 30 seconds; consequently, they experienced the initial tingling sensation associated with real stimulation but insufficient current for any neuronal modulation. As such, participants were blinded to the stimulation condition they were experiencing and, anecdotally, did not accurately report which session provided active stimulation. Previous studies in the literature report effective blinding with the use of 1 mA tDCS [25] and clinical trials using 2 mA also assume adequate blinding [26], [27].

**Figure 1:**
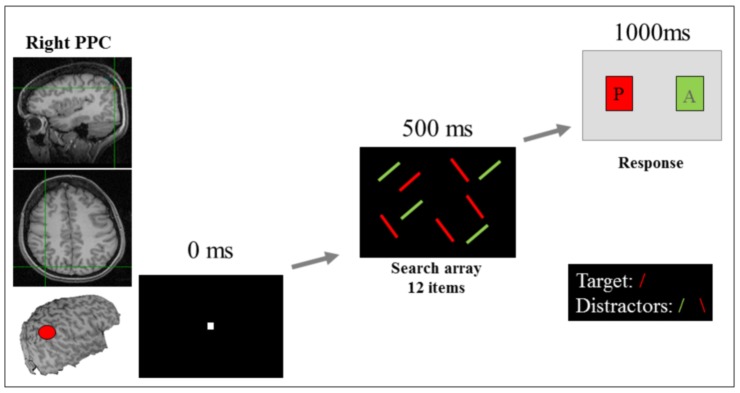
Experimental site and task. Participants had to search for a forward red slash amongst red backslashes and green slashes as quickly and as accurately as possible.

The rPPC location was measured as being 9 cm dorsal and 6 cm lateral to the right of the mastoid-inion, as this corresponds with the angular gyrus known for its role in visual search tasks as demonstrated using TMS [9], [28]. The location of this site is shown in Figure 1. The area of stimulation was defined by the size of the electrodes [29] with maximum current being discharged directly below the electrodes [30], thus, precise functional localisation of the sites of interest was not necessary and centring the electrode over the known regions was sufficient.

tDCS was applied in a dimly lit room adjacent to the scanning control room with no sensory stimuli occurring in the background. The participant was seated comfortably with their eyes closed, and talking and moving was discouraged (unless there was a problem). Earplugs (necessary for the fMRI scanning) were applied prior to the start of tDCS.

### fMRI

At the end of the tDCS period (15 minutes) participants were guided into the scanner room and their head placed within the head coil. This procedure took less than 4 minutes for each participant. Functional MRI scanning was started immediately with the first visual search block occurring within 5 minutes (mean time: 295.68 s±37.22 s) of the end of the tDCS period.

### Visual Search Task

In the MR scanner, stimuli were delivered using the E-Prime 2.0 (Psychology Software Tools Inc., Pittsburgh, PA, USA) software package (Neurobehavioral Systems) run on an IBM compatible personal computer. Participants viewed the stimuli by looking directly upwards at a mirror directed at a monitor (Cambridge Research Systems Ltd. BOLD screen MR Safe display; 1920×1200 resolution, refresh rate 60 Hz) which was placed behind the bore of the MRI scanner. The experiment was completed in a dark room.

The search arrays consisted of red and green lines on a black background (Figure 1). The target was always a red forward slash (oriented at 45° from vertical), and distractors were green forward slashes and red backslashes (oriented at −45° from vertical). Search arrays contained 12 items: in target present trials there was one target and 11 distractors (five red backslashes and six green forward slashes), and in target absent trials there were 12 distractors (six red backslashes and six green forward slashes). The target was present on 50% of trials, with the target appearing on the left and right side of the array equally frequently. Each line measured 2.5° of visual angle in length and 0.4° of visual angle in width. The whole screen measured 32° of visual angle horizontally and 24° vertically. The 12 items in each search array were randomly placed into a 10×6 virtual grid to prevent items from overlapping.

### Visual Search Procedure

At the beginning of each trial a white fixation cross (0.5° of visual angle) was presented centrally for 500 ms, followed by the presentation of a search array. Participants had to decide as quickly and as accurately as possible whether the target was present or absent, and make the corresponding key-press response (MRI compatible button box, Psychology Software Tools Inc., Pittsburgh, PA, USA). The search array remained on the screen up to a maximum of 2000 ms. If participants responded before that then a blank screen was presented for the remainder of the time, allowing total trial duration to be independent of response time. Following this, a blank screen was then presented for a variable duration (from 3000 ms to 5000 ms) before the next trial was initiated. No feedback was provided about the accuracy of the response. Participants completed two blocks of visual search trials (90 target present and 90 target absent trials per block), each block taking 13 min 50 sec to complete. Upon completion of block 1, there was a 15 minute break in which a structural scan was carried out followed by block 2 of visual search trials.

### fMRI Data Acquisition

All scans were performed on a 3 T Magnetom Trio MR scanner (Siemens Medical Systems, Erlangen, Germany) using standard gradients and a 32 channel head coil.

For each experimental block of each subject, one series of 390 functional volumes of T2*-weighted axial EPI-scans including five initial dummy scans, which were discarded immediately, was acquired parallel to the AC/PC line with the following parameters: number of slices (NS): 35; slice thickness (ST): 3.0 mm; interslice gap (IG): 0.3 mm; matrix size (MS): 96×96; field of view (FOV): 212×212 mm; echo time (TE): 30 ms; repetition time (TR): 2160 ms; flip angle (FA): 90°. For each participant an anatomical scan was acquired using a high-resolution T1-weighted 3D-sequence (NS: 192; ST: 1 mm; MS: 512×512; FOV: 256×256 mm; TE: 2.52 ms; TR: 2250 ms; FA 9°).

### fMRI Data Analysis

MR images were analyzed using Statistical Parametric Mapping (SPM8; www.fil.ion.ucl.ac.uk) implemented in MATLAB R2010b (Mathworks). All images were realigned to the first image to correct for head movement. Unwarping was used to correct for the interaction of susceptibility artifacts and head movement. After realignment and unwarping, the signal measured in each slice was shifted in time relative to the acquisition time of the middle slice using a sinc interpolation to correct for their different acquisition times. Volumes were then normalized into standard stereotaxic anatomical MNI-space by using the transformation matrix calculated from the first EPI-scan of each subject and the EPI-template. The default settings for normalization in SPM8 with 16 nonlinear iterations and the standard EPI template supplied with SPM8 were used. Afterward, the normalized data with a resliced voxel size of 3 mm×3 mm×3 mm were smoothed with a 8 mm FWHM isotropic Gaussian kernel to accommodate intersubject variation in brain anatomy. The time series data were high-pass filtered with a high-pass cutoff of 1/128 Hz. The first-order autocorrelations of the data were estimated and corrected for.

For each of the two scanning sessions (tDCS, sham) and each of the two blocks of the visual search task, four conditions were modelled in the analyses: correct responses to targets, incorrect responses to targets, correct responses to non-targets, incorrect responses to non-targets. The expected hemodynamic response at stimulus onset was modelled by two response functions, a canonical hemodynamic response function [31] and its temporal derivative. The temporal derivative was included in the model to account for the residual variance resulting from small temporal differences in the onset of the hemodynamic response, which is not explained by the canonical HRF alone. The functions were convolved with the event-train of stimulus onsets to create covariates in a general linear model. Subsequently, parameter estimates of the HRF regressor for each of the different conditions were calculated from the least mean squares fit of the model to the time series. Parameter estimates for the temporal derivative were not further considered in any contrast.

For the group analysis, only parameter estimates for correct target present trials within each scanning session/task block were considered. An SPM8 random-effects group analysis was performed by entering parameter estimates for all subjects into a within-subject one-way ANOVA, in which subjects are treated as random variables.

We report all functional activations at an uncorrected significance level of p<0.005. To correct for multiple comparisons across the whole brain, a Monte-Carlo simulation of the brain volume was employed to establish an appropriate voxel contiguity threshold [20]. This correction has the advantage of higher sensitivity, while still correcting for multiple comparisons across the whole brain volume. Assuming an individual voxel type I error of p<0.005, a cluster extent of 20 contiguous resampled voxels was indicated as necessary to correct for multiple voxel comparisons across the whole brain at p<0.01 (based on 10,000 simulations).

The reported voxel coordinates of activation peaks were transformed from MNI space to Talairach and Tournoux atlas space [32] by nonlinear transformations. The respective Matlab code can be found at http://imaging.mrc-cbu.cam.ac.uk/downloads/MNI2tal/mni2tal.m. This was done to allow the use of the Talairach and Tournoux atlas to identify the anatomical brain regions for the activation peaks. Anatomical localization of activations was automatically assessed using the MNI Space utility (MSU; http://www.ihb.spb.ru/~pet_lab/msu/msumain.html).

## Results

Mean accuracy was above 95% in all conditions and there were no significant differences between conditions (p>.05). Analyses were restricted to correct target present responses.

### The Effect of tDCS on Performance in the Visual Search Task

**Figure 2:**
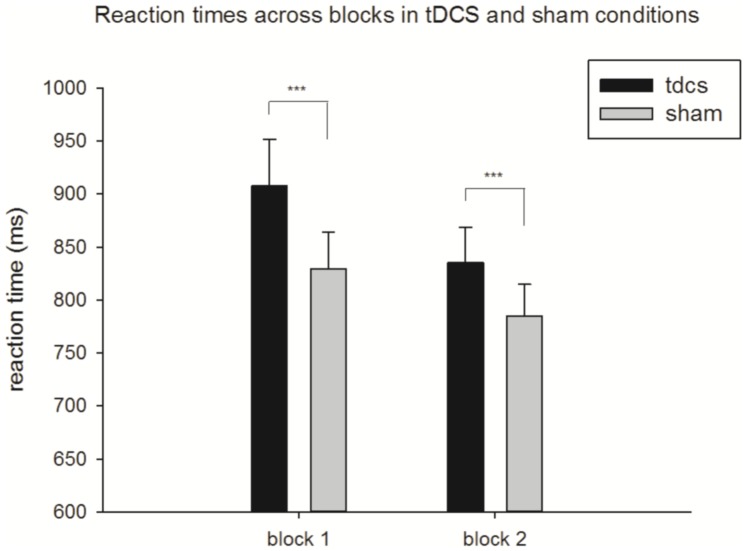
Reaction times for tDCS and sham conditions in Blocks 1 & 2. ***denotes significance to p<0.001.

A two factor (Stimulation [tDCS, sham]×Block [1], [2]) repeated measures ANOVA revealed significant main effects of Stimulation (F_(1,10)_ = 25.488, p = 0.001) and of Block (F_(1,10)_ = 18.488, p = 0.002). There was no significant interaction between Stimulation and Block (F_(1,10)_ = 3.163, p = 0.160). Pairwise t-tests further investigating these differences showed a significant increase in reaction time between tDCS and sham stimulation in both block 1 (t = 3.766, df = 19, p = 0.001) and block 2 (t = 4.255, df = 19, p = 0.001). As Figure 2 shows, even though reaction times were overall faster in block 2, the tDCS effect on reaction time was still maintained.

### The Effect of tDCS on Neural Substrates Involved in the Visual Search Task

**Figure 3:**
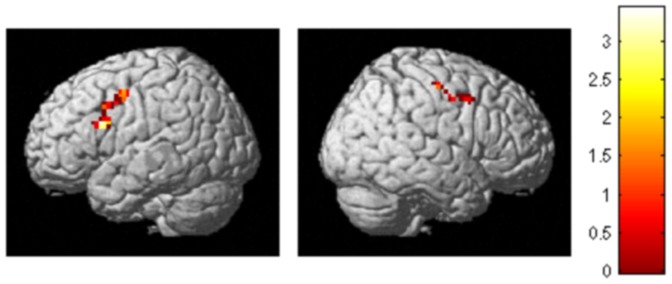
Brain regions showing a stronger activation for correct target present trials after sham tDCS as compared to tDCS stimulation (p<0.01, corrected for multiple comparisons).

To delineate the influence of cathodal tDCS on functional brain activations during the visual search task, we compared activation for correct target present trials after tDCS application to those after sham tDCS. As can be seen from Figure 3, during the first block of the visual search task (which started approximately five minutes after the end of the stimulation period) a number of frontal lobe regions were significantly less active following cathodal tDCS as compared to sham tDCS. Within the left hemisphere, the peak activation difference was located at Talairach and Tournoux coordinate [53, 10, 36] (Brodmann area [BA] 9) which is located in the left middle frontal gyrus. The activation cluster also extended into the left precentral (BA 6) and inferior frontal gyri (BA 44). In the right hemisphere, the peak of the activation lay in the right postcentral gyrus at Talairach and Tournoux coordinate [−53, −13,51] (BA 3). This activation cluster also extended into the right middle gyrus (BA 8) and slightly into the precentral gyrus (BA 6). However, there was no significant difference between the tDCS and the sham stimulation during the second block of the visual search task, which began approximately 34 minutes after the end of the stimulation period.

There was significant BOLD signal of rPPC during the visual search task in both sham and tDCS (45, −39, 58) conditions, and although there is less activity in rPPC following tDCS, this does not manifest as a significant difference to that seen in the sham condition.

## Discussion

The aim of this experiment was to examine the contingency of activity between rPPC and frontal regions such as the frontal eye fields during conjunction visual search tasks. fMRI activations reveal that when electrical activity in rPPC is decreased (via cathodal tDCS), there is significantly less BOLD response in frontal regions than seen in the same areas when sham tDCS is applied. Also, this effect is bilateral. This data not only suggests that there is a functional coupling between rPPC and frontal regions, but also that rPPC communicates with both prefrontal cortices.

Prefrontal regions involved in visual search include frontal eye fields (FEF) and associated cortex involved in the orientation of attention [6], [7], [14], [33]. The finding that prefrontal activity is altered following disruption elsewhere within the fronto-parietal network is, however, novel. One explanation is that activity in regions involved in saccadic control and attentional selection including FEF (BA8, the activity of which was modulated in this experiment in the right hemisphere), in addition to regions involved in decision making (BA9, activity modulation seen here in the left hemisphere), is selectively depressed leading to increases in reaction time following cathodal tDCS. Whilst it has been established using TMS that both right and left FEF have critical roles in visual search [13], only activity in right FEF in this experiment is affected by cathodal tDCS over rPPC. This finding may reveal a more robust information flow between rPPC and right FEF that does not exist between rPPC and left FEF. Indeed, there are differences in the specificity of involvement between right and left FEF in visual search, with right FEF engagement even in trials where the target location has been primed which is not evident with left FEF [13]. It is therefore plausible to assume that left FEF involvement in visual search may belong to a communication network separate to that seen between rPPC and right FEF.

Given the relative timing of involvement of FEF and PPC in conjunction visual search tasks, one could assume that the direction of communication is the reverse. Using single or double pulse TMS, it has been established that FEF are critically involved in the processing of a visual search task 80 ms earlier than rPPC [11], [14]. This is consistent with their purported roles, i.e. that FEF is involved in target detection (perhaps via communication with extrastriate cortex), whilst rPPC is important for the translation of visual information into action [11], [12], [34].

However, a recent study by Ball et al. (2013) revealed no effect of anodal or cathodal tDCS on conjunction visual search performance when applied to right FEF, whilst cathodal stimulation over rPPC impaired performance (an effect replicated in the present study). This may indicate a transient but dynamic role of right FEF in the processing of this task, one which is non-amenable to disruption by tDCS. It may also mean that if right FEF is disrupted, left FEF can compensate. However, the experimental design used discounts the possibility that contingent information flows from right FEF to rPPC. If this was the case then prolonged disruption of right FEF would affect rPPC activity, thereby impairing performance in the same manner as seen when tDCS was applied directly to rPPC. The current study provides evidence to confirm this directionality of information required at least for our performance indicator as there is a contingent task related decrease of activity between rPPC and prefrontal regions.

Other prefrontal activation changes can be accounted for by the coupling of these regions with rPPC with respect to the visuomotor transformation required to respond to the presence or absence of a target. Visual search tasks are a useful tool for investigating not only visual selection and attentional aspects of search, but also the action resulting from such processing (the response indicator, in this case, a button press). rPPC has previously been shown to be critical in this aspect of such tasks [11], [12] and therefore it is reasonable to suggest that cathodal tDCS over rPPC may affect premotor regions involved in the response phase of visual search. In this experiment only the right hand was used for responding, and so this argument is further supported by the left precentral regions, involved in complex and coordinated movements (BA6), being preferentially affected. Contingent decreased activity in left BA44 also lends evidence to the recent argument that Broca’s area has a role to play in exerting control over cognitive processes [35]. It is interesting to note that the reaction time deficits seen following cathodal tDCS over rPPC might be due to depression of activity in such a widespread number of cortical areas.

In addition to its roles in spatial orientation of attention and visuomotor transformations, it has been established, using a change blindness paradigm, that rPPC plays a critical role in visual short term working memory [36], [37]. Indeed, theta burst TMS has also established that rPPC and frontal regions may have dissociable roles in this respect with rPPC involvement evident in spatial or orientation working memory and left inferior frontal gyrus (BA 44) required for working memory for colour or identity [38]. Since our task required a conjunction of orientation and colour, this may add further understanding to why a decrease in activity over rPPC led to a concomitant decrease of activity in left inferior frontal gyrus as these regions may be required to work together for the efficient processing of any working memory component of the task. However, it would seem that in a task such as that used in this experiment in which the target identity remains stable, the importance of the prefrontal working memory component diminishes [39] with lesions of the prefrontal cortex having little effect on visual search tasks in which the target remains stable [40].

Decreases in post-central activation in the right hemisphere are a curiosity. One may expect to see increased activation in somatosensory regions following tDCS due to the sensory experience associated with the technique, although this should be equivalent in sham conditions. Also, given that these modulations were seen in the right hemisphere it is reasonable to assume that their cause originated on the left side of the body, where the reference electrode was placed. A decrease in activity might be explained by a lingering BOLD signal indication of habituation to the tingling that is initially more evident under the frontal electrode due to the lack of hair there. There is a behavioural habituation; participants rarely report tingling after the first 30–60 seconds post stimulation onset (thus enabling a strong sham condition). It is possible that the decrease in activation in right post-central cortex is a neurological manifestation of such behavioural habituation.

The changes in neural activity seen in this experiment are task related as they are seen in the tDCS condition when compared with a sham condition. However, the BOLD effect differences were only evident for the first block of visual search trials and this was mediated by a more comparable BOLD effect between sham and tDCS conditions in block 2. The second block of visual search trials began 29 minutes after the commencement of block 1, which was approximately 34 minutes after the end of the tDCS period. Although tDCS had a behavioural effect (evidenced by a significant lengthening of reaction times) there was no modulation of the BOLD signal during the second block. It may be that changes in activity of frontal regions contingent on activity from rPPC is dynamic soon after tDCS, thereby resulting in BOLD response changes. The depressed information flow from rPPC immediately after tDCS would seem to reduce the involvement of prefrontal regions in the processing of conjunction visual search. Neuromodulation by cathodal stimulation is mediated by a reduction in glutamate concentration [41] with offline effects driven by synaptic modulation [42]. It is reasonable to assume that 35 minutes following the tDCS session, these changes in activity in the brain are not sufficient to drive a change in BOLD response even though a behavioural effect with respect to sham was still in evidence.

A similar conundrum exists as to why no differences in BOLD signal between the sham and tDCS conditions were seen in rPPC, where the cathode was placed. To clarify, there is significant BOLD signal in rPPC during the visual search task in both conditions, and although there is less activity in rPPC following tDCS, this does not manifest as a significant difference to that seen in the sham condition. However, previous research in which cathodal tDCS was applied prior to scanning did not report significant changes in BOLD activation under the electrode either, rather it reported effects in task dependant associated cortex, albeit not over the distance in cortex that we did [43]. Again, in this case, it may be that decreasing electrical activity in an area of the cortex, whilst causing a behavioural effect, does not result in changes to a relatively conservative measure such as blood flow to that particular area. Since rPPC would still be engaged to some degree (albeit not as efficiently) in the processing of the task, it seems reasonable to assume that the requirement of oxygen to this region may not significantly change. This view makes the contingent decrease in activity in frontal regions leading to a decrease in blood flow to these regions all the more striking.

Evidence from studies using magnetoencephalography confirm that long term changes to the alpha and gamma band are apparent up to 35 minutes following tDCS, and that this is indicative of within-network modulations [44]. It cannot be discounted that the rPPC effect in this experiment, and previous experiments which have demonstrated behavioural effects with tDCS, may actually be manifested by downstream modulations of the frontal regions which have been indicated in the current experiment.

## Conclusions

We have demonstrated a functional communication between rPPC and bilateral prefrontal regions including FEF, and have ascertained that prefrontal activations are contingent upon information flow from parietal cortex during a conjunction visual search task.
